# Preparation of Antimicrobial Hyaluronic Acid/Quaternized Chitosan Hydrogels for the Promotion of Seawater-Immersion Wound Healing

**DOI:** 10.3389/fbioe.2019.00360

**Published:** 2019-12-10

**Authors:** Xinlu Wang, Pengcheng Xu, Zexin Yao, Qi Fang, Longbao Feng, Rui Guo, Biao Cheng

**Affiliations:** ^1^The First Clinical Hospital of Guangzhou Medical University, Guangzhou, China; ^2^Department of Plastic Surgery, General Hospital of Southern Theater Command, PLA, Guangzhou, China; ^3^Department of Public Health, Guangdong Pharmaceutical University, Guangzhou, China; ^4^Beogene Biotech (Guangzhou) Co., Ltd., Guangzhou, China; ^5^Key Laboratory of Biomaterials of Guangdong Higher Education Institutes, Department of Biomedical Engineering, Guangdong Provincial Engineering and Technological Research Center for Drug Carrier Development, Jinan University, Guangzhou, China

**Keywords:** quaternized chitosan, hydrogel, hyaluronic acid, seawater immersion, wound healing

## Abstract

Wound immersion in seawater with high salt, high sodium, and a high abundance of pathogenic bacteria, especially gram-negative bacteria, can cause serious infections and difficulties in wound repair. The present study aimed to prepare a composite hydrogel composed of hyaluronic acid (HA) and quaternized chitosan (QCS) that may promote wound healing of seawater-immersed wounds and prevent bacterial infection. Based on dynamic Schiff base linkage, hydrogel was prepared by mixing oxidized hyaluronic acid (OHA) and hyaluronic acid-hydrazide (HA-ADH) under physiological conditions. With the addition of quaternized chitosan, oxidized hyaluronic acid/hyaluronic acid-hydrazide/quaternized chitosan (OHA/HA-ADH/O-HACC and OHA/HA-ADH/N-HACC) composite hydrogels with good swelling properties and mechanical properties, appropriate water vapor transmission rates (WVTR), and excellent stability were prepared. The biocompatibility of the hydrogels was demonstrated by *in vitro* fibroblast L929 cell culture study. The results of *in vitro* and *in vivo* studies revealed that the prepared antibacterial hydrogels could largely inhibit bacterial growth. The *in vivo* study further demonstrated that the antibacterial hydrogels exhibited high repair efficiencies in a seawater-immersed wound defect model. In addition, the antibacterial hydrogels decreased pro-inflammatory factors (TNF-α, IL-1β, and IL-6) but enhanced anti-inflammatory factors (TGF-β1) in wound. This work indicates that the prepared antibacterial composite hydrogels have great potential in chronic wound healing applications, such as severe wound cure and treatment of open trauma infections.

## Introduction

With the extensive use of high-tech weapons, especially precision-guided missiles on the battlefield, the trauma and burns caused by high-energy explosion in naval battle have become the most important and challenging healthcare issues (Zhu et al., 2017; Chen et al., 2019). In modern high-tech naval warfare, the unavoidable exposure of open traumas to seawater with high salt, high sodium, and a high abundance of pathogenic bacteria, especially gram-negative bacteria, can cause serious infections and wound-repair difficulties (Zhang et al., 2017). Current traditional antimicrobials such as antibiotics, iodine, silver, and zinc oxide are effective in preventing bacterial infection of skin wounds, but they cause resistance to last-line antibiotics and cause significant damage to vital organs (Chung et al., 2017; Lin et al., 2019). Considering these imperfect treatments, the development of new and more effective antimicrobials is still highly desired in clinical application.

Chitosan is a kind of natural polymer that has the characteristics of biodegradability, biocompatibility, and antimicrobial activity (Thattaruparambil et al., 2016; Liang et al., 2019a). It meets the requirements of environmental protection and has become one of the research hotspots in the development of natural antimicrobial agents (Sarhan et al., 2016). The antimicrobial mechanism of chitosan is based on the positive charge of the amino group at the C-2 position after protonation at a pH below 6, which can interact with the surface of bacteria and cause bacterial death (Pires et al., 2013; Yildirim-Aksoy and Beck, 2017). However, chitosan is insoluble in neutral and alkaline aqueous solutions with pH values of >6.5, which greatly limits its application (Mohamed et al., 2017). Recently, quaternized chitosan (QCS) has received more attention from researchers, as it can replace traditional antimicrobials and reduce organ damage (Thanou et al., 2002; You et al., 2016). The addition of quaternary amino groups in chitosan greatly enhances the water solubility of chitosan, and quaternized chitosan with antimicrobial activity combines with polyatomic amino groups to form double antimicrobial active groups, which greatly improves its antimicrobial field of application.

In wound treatment, wound dressing materials with superior properties are typically used to facilitate wound healing, of which hydrogels that have high water content, flexible mechanical properties, and good biocompatibility are considered promising candidates for practical application (Li et al., 2018, 2019; Yi et al., 2018). Firstly, by providing a porous structure and having a suitable swelling ratio, a hydrogel matrix can allow the presence of oxygen, remove wound exudates, and maintain a moist wound bed to promote wound healing (Kaoru et al., 2010; Rakhshaei and Namazi, 2017). Secondly, the antibacterial property of traditional dressing is endowed by antibiotics capsulated in the hydrogel matrix (Li et al., 2016). However, hydrogels with inherent antimicrobial properties have received widespread interest among biomaterial researchers (Gonzálezhenríquez et al., 2017; Zhao et al., 2017; Kumar et al., 2018). Thirdly, unlike traditional wound dressings (gauze and cotton wool), biodegraded hydrogel dressings are easy to peel off and degrade spontaneously, which avoids pain and secondary trauma during dressing changes (Yang et al., 2018).

Inspired by the concept of moist wound healing, numerous novel hydrogels have been designed, and these play an important role in the treatment of various wounds (Blacklow et al., 2019; Wang et al., 2019). The majority of hydrogels are prepared from natural polymer materials (e.g., alginate, carboxymethylcellulose, dextran, gelatin, collagen, and hyaluronic acid) and synthetic polymer materials [e.g., methoxy polyethylene glycol, poly(vinyl alcohol), peptide, and polyamidoamine] because of their excellent biocompatibility and biodegradability (Travan et al., 2016). Hyaluronic acid (HA), the main component of the extracellular membrane (ECM), can increase cell–matrix interaction and initiate the signal transduction essential for cell survival and function and has been widely used in the biomedical materials field because of its property of easily peeling, excellent biocompatibility, and high water retention (Purcell et al., 2012; Julia et al., 2013; Zhu et al., 2018a; Liang et al., 2019b).

**Scheme 1:**
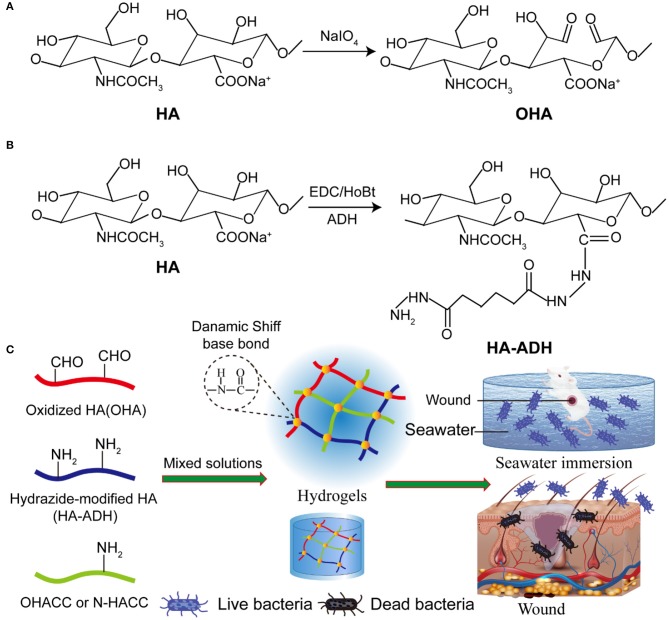
The Davies-ENDOR pulse sequence. Schematic representation of hydrogel synthesis **(A)**. Steps of OHA synthesis. **(B)** Steps of HA-ADH synthesis. **(C)** Schematic representation of the preparation of the OHA/HA-ADH/O-HACC and OHA/HA-ADH/N-HACC hydrogels.

In this work, biocompatible hydrogel wound dressings with inherent antibacterial properties were prepared by dynamic Schiff base linkage (Scheme 1). We furthermore demonstrated that these hydrogel dressings greatly promoted the healing process in a seawater-immersed full-thickness skin defect model. The hydrogels were prepared by mixing quaternized chitosan/hyaluronic acid-hydrazide (HA-ADH) solutions and oxidized hyaluronic acid (OHA) solution under physiological conditions. Hyaluronic acid (HA) was chosen as a hydrogel substrate due to its advantageous properties of high water retention performance and good biocompatibility (Li et al., 2017; Park et al., 2019). In this work, the addition of quaternized chitosan enhanced the mechanical properties of OHA/HA-ADH hydrogel. The hydrogels exhibited excellent antibacterial performance compared to the previous reported antibacterial hydrogels *in vitro* and *in vivo*. Furthermore, the results for the wound contraction area, bacteria in wound, histopathological examinations, collagen analysis, and pro-inflammatory factors (TNF-α, IL-1β, and IL-6) and anti-inflammatory factors (TGF-β1) in wound were employed to evaluate the *in vivo* therapeutic effect. The results indicated that these antibacterial hydrogels have good biocompatibility and show great potential as wound dressings, especially for the healing of severe wounds and open trauma infections.

## Materials and Methods

### Reagents and Materials

Chitosan (CS, Mw = 3 kDa, degree of deacetylation = 95%) was obtained from Nantong Lushen Bioengineering Co., Ltd. (Jiangsu, China). Benzaldehyde, Glycidyltrimethylammonium chloride (GTMAC), (3-chloro-2-hydroxypropyl) trimethyl-ammonium chloride S, and ethylene glycol was purchased from Sinopharm Chemical Reagent Co., Ltd. (Shanghai, China). Hyaluronic acid (HA, Mw = 200 kDa) was purchased from Bloomage Freda Biopharm Co., Ltd. (Shangdong, China). Adipic dihydrazide (ADH), hydroxy-benzotriazole (HOBt), and dimethyl sulfoxide (DMSO) were purchased from Aladdin Chemical Company (Shanghai, China). Sodium periodate, 1-ethyl-3-(3-dimethylaminopropyl)-carbodiiminde (EDC), and hyaluronidase were obtained from Shanghai Yuanye Bio-Technology Co., Ltd. (Shanghai, China). The organic silicon film (BD film KYQ-500) was purchased from Hangzhou Baoerde New Materials Technology Co., Ltd (Hangzhou, China).

The L929 fibroblast cell line was obtained from Beogene Biotechnology Co., Ltd. (Guangzhou, China). Cell Counting Kit-8 (CCK8) was obtained from Beyotime Biotechnology Co., Ltd. (Shanghai, China). Live/dead cell staining kits were purchased from BestBio Bio-Technology Co., Ltd. (Shanghai, China). The bacteria strains of *Escherichia coli* (*E. coli*, CMCCB 44102) and *Staphylococcus aureus* (*S. aureus*, CMCCB 26003) were purchased from Guangdong Institute of Microbiology (Guangzhou, Guangdong). VRBA-MUG selection medium, Mannitol salt agar, and Pseudomonas agar base/CN-AGAR were obtained from HuanKai Microbial Biotechnology Co., Ltd (Guangzhou, China). The other reagents were listed as follows: Dulbecco's modified Eagle's medium (DMEM, Gibco, USA), fetal bovine serum (FBS, HyClone, USA), trypsin (Amresco, USA), and dimethyl sulfoxide (DMSO, Sigma-Aldrich, USA). All other reagents were analytical grade unless otherwise noted.

### Synthesis of Quaternized Chitosan (QCS)

**Scheme 2:**
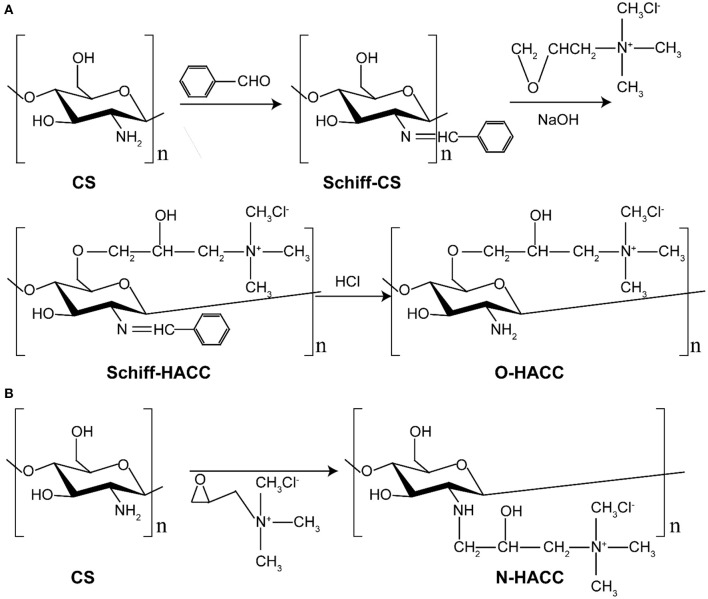
**(A)** Steps of O-HACC polymer synthesis. **(B)** Steps of N-AACC polymer synthesis.

O-HACC and N-HACC were synthesized according to the previous literature with slight modifications (Hu et al., 2010; Xin et al., 2015; Mohamed et al., 2017). The synthesis route of O-HACC and N-HACC polymer is presented in Scheme 2.

#### Synthesis of O-HACC

Briefly, 0.3 g of chitosan powder was dissolved in 120 mL of 10% acetic acid for 4 h, 40 mL ethanol was added, and then 15.8 g benzaldehyde was added to the mixed solutions with continuous stirring. After the mixed solutions had been heated and reacted at 60°C for 20 h, the pH was adjusted to pH 7.0 using NaOH solution. The mixture was precipitated, filtered, and washed fully with methanol. The yellow powder chitosan imine Schiff base (Schiff-C S) was obtained by vacuum drying. A quantity of 2.5 g chitosan imine Schiff base and 7.5 g 2,3-epoxypropyl trimethylammonium chloride (ETA) were placed in a straight glass cylinder. Then, 0.02 g NaOH was dissolved in 10 mL water. The mixture was dissolved with the 10 mL 0.2 wt% NaOH solution with continuous stirring to obtain a viscous crude product. The product was precipitated by ethanol, filtered, and washed with 80% ethanol aqueous solution. The crude product was extracted by Soxhlet extractor with absolute ethyl alcohol as the solvent for 24 h and dried in a vacuum. The solid powder product was obtained and named O-HACC. The degree of quaternization (DQ) of the obtained O-HACC was 65.2%.

#### Synthesis of N-HACC

Briefly, 3.0 g of chitosan powder was suspended in 27 mL of isopropanol for a 4-h period at 8°C, and then 35 wt.% GTMAC was added dropwise to the suspension with continuous stirring. The pH of the mixed solutions was then adjusted to pH 7.0 using NaOH solution. After reaction at 80°C for 7 h, the mixture was poured into cold acetone, and stirring was continued for 12 h at 4°C. The mixture was washed three times with cold acetone and dialyzed in a dialysis sack (8–12 KDa molecular weight (MW) cut-off) for 3 days. The purified product was obtained by overnight lyophilization of the dialysate, and the fine powder collected was named N-HACC. The degree of quaternization (DQ) of the N-HACC obtained was 98.4%.

### Characterization of O-HACC and N-HACC

#### Water Solubility

Chitosan (20 mg) was dissolved in 2 mL distilled water and 2 mL 1% (w/v) acetic acid solution at 25°C for 2 h with constant stirring, respectively. O-HACC (20 mg) and N-HACC (20 mg) was dissolved in 2 mL distilled water at 25°C for 2 h with constant stirring, respectively. The clarity of the solution was examined and photographed.

#### pH Dependence of the Water Solubility

The solubility was assessed for the pH range 3.0–12.0 via the turbidimetric titration method. O-HACC (40 mg) and N-HACC (40 mg) were dissolved in 20 mL of 1% acetic acid, and the solution was gradually titrated (20 μL) with NaOH (1 mol/L) to the final pH, with constant stirring. Absorbance measurements of the solutions were recorded using a UV spectrophotometer (UV-2550, Shimadzu, Tokyo, Japan) at λ = 600 nm.

#### Characterization of ^1^H NMR and FT-IR

The chemical structure of the synthetic O-HACC and N-HACC was performed by ^1^H NMR (AVANCE III 600M, Bruker, Germany). FT-IR spectral data were recorded with a Fourier transform-infrared spectrometer (FT-IR; Spectrum One, Perkin Elmer, Norwalk, USA). The samples were pressed with KBr.

#### Minimum Inhibitory Concentration (MIC) Measurements

The MIC of O-HACC and N-HACC were determined using a broth microdilution method, as described previously (Chin et al., 2018). Briefly, 50 μL deionized water solution of O-HACC and N-HACC with different concentrations was placed into each well of a 96-well microplate. Then, 50 μL of bacterial TSB solution (3 × 10^5^ CFU/mL) was added into each well containing the polymer solution. A bacterial TSB solution without O-HACC and N-HACC was used as the control. The 96-well plate was kept in an incubator at 37°C under constant shaking at 100 rpm for 18 h. The MIC was taken as the concentration of the polymer at which no microbial growth was observed with unaided eyes and with the microplate reader at the end of 18-h incubation. All measurements were repeated three times in the same assay plate.

### Preparation of the Antimicrobial Composite Hydrogels

#### Oxidized HA Synthesis (OHA)

OHA was synthesized according to a previously reported method, with a slight modification (Taichi et al., 2007). HA (1 g, 2.48 mmol) was dissolved in 150 mL distilled water, and then 5 mL NaIO_4_ solution (0.5 mol/L) was added. The mixed solution was stirred at room temperature for 2 h, protected from light. The reaction was terminated by adding 1 mL ethylene glycol and stirring for an additional 1 h. The solution was dialyzed in a dialysis sack (MWCO 8,000–15,000) for 3 days against distilled water, changing the water three times per day, and then OHA was obtained by freeze-drying.

#### Hydrazide-Modified HA Synthesis (HA-ADH)

Hydrazide-modified HA (HA-ADH) was synthesized according to a previously established carbodiimide chemical process (Jia et al., 2004). In brief, HA (500 mg, 1.24 mmol) was dissolved in 125 mL distilled water, and then 8 g ADH was added. Subsequently, 10 mL DMSO/H_2_O solution (V:V = 1:1) with 750 mg EDC and 660 mg HoBt was added into the previous HA solution, and the HA was reacted with ADH at a pH 4.75 for 4 h. The reaction was terminated by increasing the solution pH to 7.5 by adding NaOH solution. The HA–ADH solution was precipitated in ethanol, and the precipitate was re-dissolved in distilled water and dialyzed in a dialysis sack (MWCO 80,00–15,000) for 3 days against distilled water, changing the water three times per day, and then HA-ADH was obtained by freeze-drying.

#### Preparation of Composite Hydrogels

The hydrogels were fabricated by dynamic chemical bonding (Schiff base) of OHA and HA-ADH in a distilled solution. For the preparation of antimicrobial composite hydrogels, 4% (w/v) OHA and 4% (w/v) HA-ADH were fully dissolved in deionized water. Subsequently, 20 mg O-HACC (or 10 mg N-HACC) was added into 0.4 mL HA-ADH solution. After thorough gentle mixing, the mixture was transferred into a 48-well plate as a cylindrical mold. The resultant hydrogels were termed OHA/HA-ADH/O-HACC (or OHA/HA-ADH/N-HACC). The whole preparation processes of OHA/HA-ADH hydrogel was the same as for OHA/HA-ADH/O-HACC except for the addition of O-HACC.

### Physical and Mechanical Properties

#### Swelling Ratio of the Hydrogels

The swelling behavior of the hydrogels was determined using the equilibrium swelling ratio (ESR). Briefly, hydrogels were weighed before the test and then immersed in phosphate-buffered saline (PBS) (pH 7.4, 37°C). At each measurement time point, the hydrogels were removed, gently blotted with filter paper to remove the excess surface water, and immediately weighed. The degree of swelling (DS) was calculated using the formula

$$\begin{array}{l} \text{DS }=\frac{W_{t}\;-\;W_{0}}{W_{0}}\;\times \;100\% \end{array}$$

where *W*_t_ and *W*_0_ are the weight of the hydrogel at time t and the weight of the hydrogel at t = 0, respectively.

#### Rheological Studies

Rheological measurements of the hydrogels were performed using a TA rheometer instrument (Kinexus, Ma Erwen instruments, Britain). For oscillatory time sweep experiments, the storage modulus (G′) and loss modulus (G″) were measured at a 10% strain, 1 Hz frequency, and 0.5 mm gap (CD mode) for 300 s. For the characterization of the linear viscoelastic range, a dynamic strain sweep test was run at frequencies ranging from 0.1 to 100 Hz.

#### Compression Test

Compression tests of the hydrogels were performed using a universal testing machine (model 5543; Instron, Norwood, MA). The hydrogel samples (8 mm in diameter and 4 mm in thickness) were prepared in advance and equilibrated in PBS. The compressive strain rate was set at 1 mm min^−1^ with a 5 N load cell under 40% constraint. The measurements were performed three times (*n* = 3).

#### Water Vapor Transmission Rate (WVTR)

The moisture permeability of the hydrogels was determined by measuring their WVTR according to the American Society for Testing and Materials (ASTM) standard. Briefly, the hydrogel samples mounted on the mouth of a cylindrical vial (diameter 9.67 mm) containing 5 mL of deionized water, and then placed into a 37°C incubator at 79% relative humidity. The WVTR of the hydrogels was calculated using the formula

$$\begin{array}{l} \text{WVTR }\left(\text{g}/{\text{m}}^{2}{\text{day}}^{-1}\right)=\frac{\Delta \text{m}}{\text{A }\times \text{ time}} \end{array}$$

where Δm is the weight of moisture loss for 24 h (g) and A is the effective transfer area (m^2^).

#### *In vitro* Degradation of the Hydrogels

The hydrogels were placed in PBS (pH 7.4) containing either 0 or 100 U/mL of hyaluronidase solution in a horizontal shaker at 37°C for 28 days. The samples were carefully removed at predetermined time intervals of 3, 7, 14, 21, and 28 days. The remaining gels were taken out, washed with distilled water, and lyophilized. The percentage of degradation of hydrogels was calculated using the formula

$$\begin{array}{l} \text{Degradation }=\frac{W_{t}}{W_{0}}\;\times 100\% \end{array}$$

where *W*_0_ is the initial weight of the freeze-dried hydrogels and *W*_*t*_ is the weight of the freeze-dried hydrogel at time t. All tests were performed on five samples (*n* = 5).

### *In vitro* Biocompatibility Test

Hydrogels pre-treated with radiation for sterilization were immersed in DMEM with 10% fetal calf serum and 1% (v/v) penicillin/streptomycin at 37°C for 24 h to obtain the leach liquor. The L929 cells were seeded on a 96-well plate at a density of 2 × 10^4^ cells per well and maintained with 100 μL of leach liquor. DMEM medium was cultured with L929 cells as controls. The leach liquor and DMEM medium were changed every 2 days. After 1, 2, and 3 days of incubation, the relative cell viabilities of the different experimental groups were measured via live/dead staining and CCK-8 colorimetric assay.

For the live/dead staining, the cells attached in each well were rinsed twice with PBS, and then 100 μL of live/dead staining stock solution was added to each well in a dark environment at 4°C for 15 min. Afterward, fluorescent images of the cells were examined using an inverted fluorescence microscope (TE2000-S, Nikon, Japan). For the CCK-8 assay, the cell culture medium was replaced by 100 μL of DMEM medium containing 10% CCK8 solution and was added to each well at 37°C for 1–1.5 h. The absorbance at 450 nm was read immediately on a microplate reader (SH1000, Corona, Japan).

### *In vitro* Antimicrobial Evaluation

A 100-μL bacteria suspension (density = 10^6^ CFU/mL) of *S. aureus* was added onto 400 μL of aseptically prepared OHA/HA-ADH, OHA/HA-ADH/O-HACC and OHA/HA-ADH/N-HACC hydrogels in 48-well plates and incubated for 1 h. Subsequently, 500 μL of LB broth was added to the hydrogels. After incubation for different durations (0, 8, and 16 h) at 37°C, the hydrogels were washed three times with PBS. The hydrogels were fixed with 4% (w/w) paraformaldehyde for 30 min. The hydrogels were further dehydrated with graded ethanol series (25, 50, 75, 90, and 100% ethanol) for 15 min each, and the samples were dried under a glass dryer. The specimens were pre-coated with gold and imaged using SEM.

### Evaluation of the Anti-seawater Immersion and Wound Healing Efficacy of the Hydrogels *in vivo*

All animal studies were approved by the Institutional Animal Care and Use Committee (IACUC) of the General Hospital of the Southern Theater Command of the PLA, and the animals were treated according to the regulations. Adult male Sprague Dawley rats (200–250 g) were used for the study *in vivo*. Fifty receptor SD rats were randomly assigned to five groups: the organic silicon film control group (group I, the negative control without seawater), organic silicon film group (group II, the negative control with seawater), OHA/HA-ADH hydrogel group (group III with seawater), OHA/HA-ADH/O-HACC hydrogel group (group IV with seawater), and OHA/HA-ADH/N-HACC hydrogel group (group V with seawater). Prior to surgery, each rat was anesthetized with 3% pentobarbital (45 mg/kg), and the dorsal surface of the rats was shaved and disinfected with iodine. Four full-thickness skin wounds (diameter = 12 mm) were then created on the right and left sides of the backbone of each rat (see Figure S1A). Later, the wounded rats were soaked in seawater at a constant temperature of 28°C for 1 h. After 1 h of seawater immersion of the full-thickness skin wounds, the wounds were sewed up with their corresponding hydrogels using silicon film (see Figure S1B).

#### Wound Closure Measurement

The wounds were photographed at the different time points on day 1, 3, 7, 10, 14, and 21 post-surgery. The wound margin was traced, and the wound size was quantified using IPP 6.0 analysis software. The wound area and wound healing rate were calculated using the formulas

$$\begin{array}{l} \text{ Wound area }=\;{\text{S}}_{\text{t}}/{\text{S}}_{0}\;\times \;100\%\\ \text{Wound healing rate }=\;\left({\text{S}}_{0}\;-{\text{S}}_{\text{t}}\right)/{\text{S}}_{0}\;\times \;100\% \end{array}$$

where S_0_ and S_t_ were the area of the original wound and the area of the wound at the testing time, respectively. Rats were sacrificed on postoperative days 3, 7, 10, 14, and 21, and the tissue including the wound site and surrounding healthy skin was excised and fixed for immunohistochemical and histological evaluations.

#### *In vivo* Evaluation of the Antimicrobial Efficacy of Hydrogels

The antimicrobial efficacy of the hydrogels was explored by homogenizing skin tissue excised on day 3 for 3 min in a 5 mL centrifuge tube containing 3 mL saline. Ten-fold serial dilutions of sample solutions were prepared, and then 100 μL samples of the diluted solution were spread onto VRBA-MUG selection medium, Mannitol salt agar, and *P. aeruginosa* selection medium, respectively. VRBA-MUG selection medium was used to selectively culture *E. coli* (Red colony morphology), Mannitol salt agar was used to selectively culture *S. aureus* (yellow-gold colony morphology), and *P. aeruginosa* selection medium was used to selectively culture *P. aeruginosa* (green-yellow colony morphology). The plates were incubated for 24 h at 37°C, and the number of colonies with 5–300 CFU was counted.

#### Histology and Collagen Staining

The excised skin tissue was fixed by immersion in 4% paraformaldehyde solution for at least 12 h. The fixed skin grafts were dehydrated with a graded series of ethanol and then dimethyl benzene and embedded in paraffin. The paraffin-embedded wounds were cut into sections with a thickness of 4 μm using a microtome (RM2016, Leica, Shanghai, China). Sections were collected and stained with haematoxylin and eosin (H&E) for histological studies and with Sirius Red for collagen detection according to routine procedures. Finally, the skin sections were photographed by a light microscope (Vectra 3, PerkinElmer, Waltham, MA). The quantifications from Sirius Red staining was performed using IPP 6.0 software. Collagen deposition in the wound sites was calculated using the formula:

$$\begin{array}{l} \text{Collagen deposition }=\;\left(\text{red }-\text{ stained area of collagen}\right)\;/\;\left(\text{tissue area}\right). \end{array}$$

#### Immunohistochemistry

For immunofluorescence staining, paraffin sections were stained using primary antibodies to TNF-a, TGF-β1, IL-1β, and IL-6 (Abcam, Cambridge, UK; 1:200 dilution). Subsequently, the sections were incubated with the universal secondary antibody (Abcam, ab205719, 1: 500 dilution) and VECTASTAIN Elite ABC reagent, washed in PBS, and reacted with DAB solution (Agilent Dako, Santa Clara, CA, USA). Finally, the nuclei were counterstained with hematoxylin. Images of the stained samples were photographed under a light microscope (Vectra 3, PerkinElmer, Waltham, MA). The positive marker percentage was quantified using IPP 6.0 software.

#### Western Blot Analysis

Tissue samples were completely homogenized in 10 times tissue volume protease inhibitors. Subsequently, the homogenates were centrifuged at 12,000 rpm for 10 min and loaded on 10–12% sodium dodecyl sulfate (SDS)-polyacrylamide gels. After being transferred to PVDF Western blot membranes, the proteins were incubated with different primary antibodies, specifically mouse anti- TGF-β1 (Abcam, ab92486, 1:1,000 dilution), rabbit anti- IL-1β (Abcam, ab108499, 1:1,000 dilution), rabbit anti-TNF-α (Abcam, ab6671, 1:1,000 dilution), and mouse anti-IL-6 (Abcam, ab9324, 1:1,000 dilution), overnight at 4°C. The membrane was further incubated with goat anti-mouse IgG H&L (HRP) conjugated secondary antibody (Abcam, ab205719, 1:5,000 dilution) for 1 h at room temperature, and was visualized via enhanced chemiluminescent reagent (Beyotime, Jiangsu, China) on X-ray film.

#### Assessment of Endotoxin and Inflammatory Mediators

Endotoxin was measured in blood serum. Blood was also obtained from each rat. The blood obtained was centrifuged at 5,000 rpm at 4°C for 10 min, and the upper serum was collected and stored at −80°C for subsequent evaluation. The serum was diluted 1:10 in sterile pyrogen-free water. The concentration of endotoxin (EU/mL) was measured by enzyme-linked immunosorbent assay (ELISA) techniques according to the manufacturer's protocol (Shanghai Biotechnology Co., Ltd., Shanghai, China). In this test, the detection limit of the endotoxin assay was 0–80 pg/mL (1 EU/mL = 100 pg/mL). The absorbance of each well was determined to be 450 nm using a microplate reader. The standard curve of endotoxin was constructed, and the corresponding concentration for the unknown sample was calculated. All determinations were performed in triplicate.

### Statistical Analysis

Data were plotted using Origin 9.1 (OriginLab Corporation, Northampton, MA, USA). Error bars represent standard deviations (SDs). The statistical trends were analyzed by one-way analysis of variance (ANOVA). ^*^p < 0.05, ^**^p < 0.01, and ^***^p < 0.001 were considered to be significantly different.

## Results and Discussion

### Synthesis of O-HACC and N-HACC

**Figure 1:**
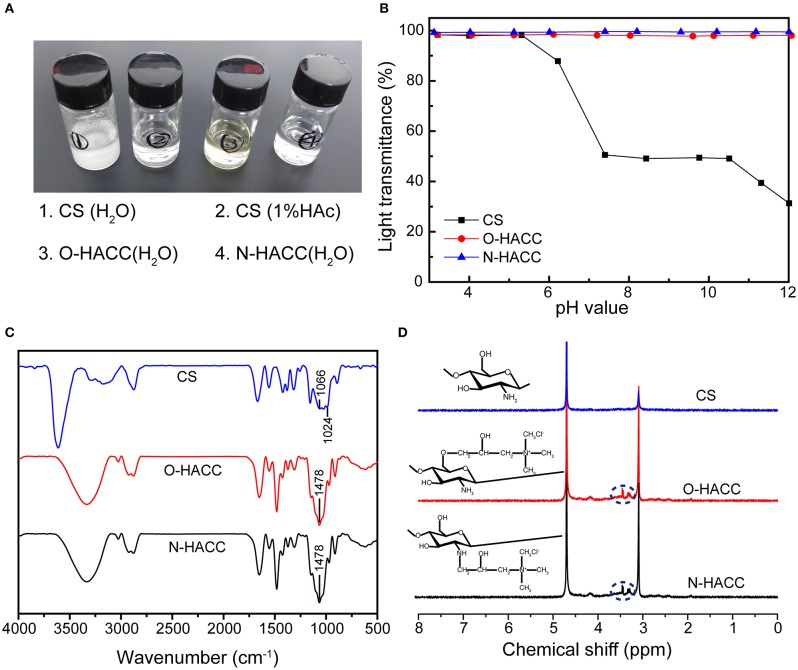
**(A)** Solutions of quaternized chitosan derivatives (water, 1.0 mg/mL) and chitosan (water and 1% HAc, 1.0 mg/mL). **(B)** Solubility of CS, O-HACC, and N-HACC at pH 3–12. **(C)** FTIR spectra, and **(D)** ^1^H NMR spectra of CS, O-HACC, and N-HACC.

Limited solubility at pH 5.5–7.4 is one of the main disadvantages of chitosan, which restricts its application in biomedicine. The CS (water and 1% HAc, 1.0 mg/mL) and O-HACC and N-HACC derivatives (water, 1.0 mg/mL) all had the appearance of solubility (Figure 1A). The dependence of the solubility of CS, O-HACC, and N-HACC on pH was further investigated (Figure 1B). CS was completely soluble in the pH range from 3 to 5, and as the pH value reached 5.0, its solubility decreased. However, O-HACC and N-HACC were almost completely soluble in the pH range from 3 to 12. The results indicated that O-HACC and N-HACC increased solubility under different pH conditions.

The FT-IR spectra of CS, O-HACC, and N-HACC are shown in Figure 1C. The main bands of chitosan included a peak at 3,303 cm^−1^, corresponding to the stretching of the O–H and N–H bonds, and the band at 1,024 cm^−1^ corresponded to the C = O bending vibration. The peak at 1,478 cm^−1^, corresponding to the C–H bending vibration of CH_3_, is presented in the infrared spectra of O-HACC and N-HACC but not that of CS (Ye et al., 2014; Xin et al., 2015). This was indicative of the quaternary ammonium salt side-chain grafted onto the CS chain. In order to further determine whether the quaternary amino bond was conjugated to CS, ^1^H NMR measurement was carried out (Figure 1D). Prior to the reaction with the quaternary amino group, the spectra of CS samples were determined. Compared to the spectra of the initial chitosan, a characteristic new peak in the strong-field region at δ = 3.2–3.5 ppm (d, -N^+^(CH_3_)_3_) indicates the presence of a trimethyl ammonium fragment in the structure of the macromolecules.

### Physical and Mechanical Properties of Hydrogels

In this study, we designed a kind of hydrogel formed by dynamic Schiff's base linkage that has an appropriate swelling ratio, good compressive properties, and a comparable modulus to human soft tissue (Chen, 2017). Following the synthesis routes shown in Schemes 1A,B, OHA and HA-ADH macromolecules were successfully obtained.

**Figure 2:**
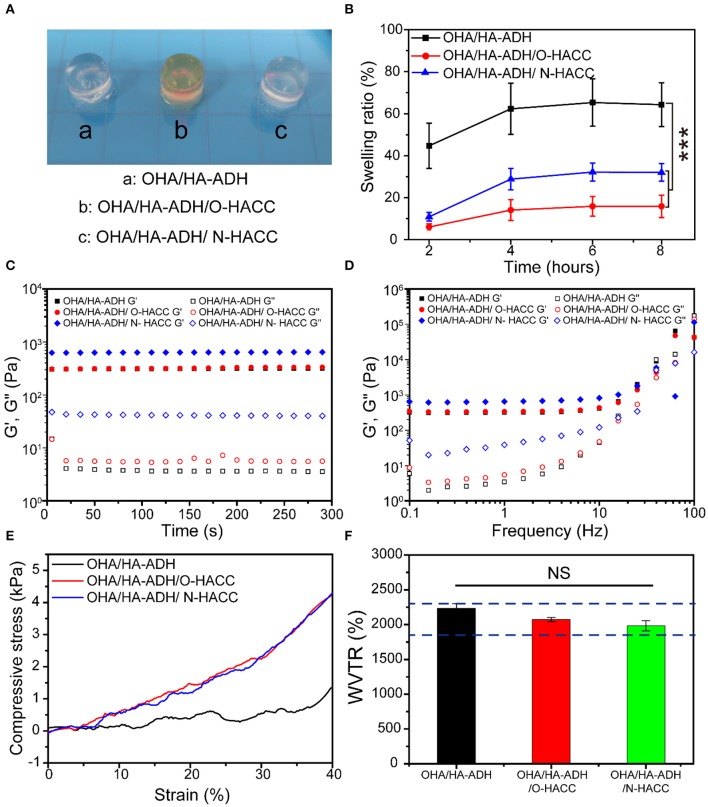
Physical and mechanical properties of the hydrogels. **(A)** Photograph of typical hydrogels. **(B)** Swelling ratio. **(C)** Rheological properties. **(D)** Gel viscosity with frequency ranging from 0.1 to 100 Hz. **(E)** Compression modulus. **(F)** Water vapor transmission rate (WVTR); the blue dotted line represents the optimal WVTR range. ***p < 0.001.

The OHA/HA-ADH and OHA/HA-ADH/N-HACC hydrogels had a white color, while OHA/HA-ADH/O-HACC had a yellow color (Figure 2A). The swelling properties of the hydrogels were investigated to illustrate their water sorption capacity (Zhou et al., 2018). The results revealed that the swelling ratios of the OHA/HA-ADH, OHA/HA-ADH/O-HACC, and OHA/HA-ADH/N-HACC hydrogels in PBS reached 44.47, 6.03, and 10.96, respectively, in the first 2 h (Figure 2B). Compared to OHA/HA-ADH hydrogels, OHA/HA-ADH/O-HACC, and OHA/HA-ADH/N-HACC hydrogels showed a lower swelling ratio, which may be related to their higher crosslink density. The hydrogels had almost reached equilibrium swelling in 4 h, with a swelling ratio in PBS of 62.31, 14.11, and 28.84, respectively. A lower swelling ratio will not have a negative impact on the organization of the material.

The gelation behavior of the hydrogels was monitored by rheological analysis. As presented in Figure 2C, G′ surpassed G″ immediately after the addition of OHA to the HA-ADH solution due to the rapid formation of the hydrogel. This could provide hydrogel formation through diffusion of the polymer solution to the surrounding tissues when injected into the body. The final G′ of OHA/HA-ADH hydrogel can reach a plateau of 10^2^-10^3^ Pa, which would be able to maintain a structurally robust 3D network shape. Meanwhile, the storage modulus (G′) of OHA/HA-ADH/O-HACC and OHA/HA-ADH/N-HACC hydrogels was increased due to an increase in hydrogel concentration and dynamic Schiff's base bonding. As shown in Figure 2D, G′ surpassed G″ with a shear frequency from 0.1 to 100 rad/s due to the Schiff's base, which indicated that the hydrogel was stable.

An ideal hydrogel should have mechanical properties that enable it to maintain its integrity during use. The compression moduli of the hydrogels are presented in Figure 2E. The results showed the OHA/HA-ADH/O-HACC and OHA/HA-ADH/N-HACC hydrogels had a higher modulus (~4 kPa) than did OHA/HA-ADH hydrogel, comparable to that of human skin (Chen, 2017). This mechanical property was related to the structure of the hydrogels, i.e., the pore size of OHA/HA-ADH/O-HACC and OHA/HA-ADH/N-HACC hydrogels, which had a higher crosslinking density, was smaller than that of OHA/HA-ADH hydrogel, leading to a more compact structure. Our results showed that the compression modulus of the hydrogel could be improved by adding O-HACC or N-HACC.

Moisture control is a critical parameter in evaluating the healing process in wounds. A higher WVTR will result in dehydration of wound tissue and scarring, while a lower WVTR will cause wound maceration and harm to surrounding tissues. The WVTR values of the OHA/HA-ADH/O-HACC and OHA/HA-ADH/N-HACC hydrogels were tested to be lower than that of OHA/HA-ADH hydrogel due to their denser network structure (Figure 2F). This may be because the addition of quaternary ammonia chitosan leads to an increase in hydrogel concentration, resulting in a denser network structure. It is reported that the WVTR of normal skin ranges from 240 to 1,920 g/m^2^·24 h, while that of an uncovered wound is in the order of 5,138 ± 202 g/m^2^·24 h (Xu et al., 2015; Yang et al., 2017). Previous studies have reported that an ideal wound dressing with a WVTR of 2,028.3 ± 237.8 g/m^2^·24 h was able to maintain a moist environment and promote exudate adsorption and that cells can also migrate more easily, promoting tissue regeneration (Xu et al., 2016). The WVTR value of the prepared hydrogels is close to that of the intact skin and ideal range, a value that avoids the risk of wound dehydration and is suitable for wound healing applications.

**Figure 3:**
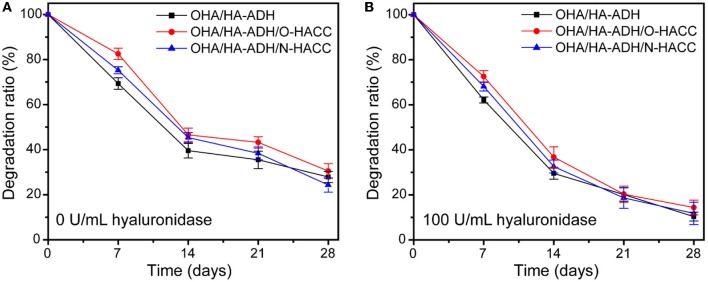
Weight loss rate of the hydrogels in pure PBS solution **(A)** and PBS solution containing 100 U/mL hyaluronidase **(B)**.

The weight loss curve of the prepared hydrogels when in PBS (either in the presence or absence of 100 U/mL hyaluronidase) decreased gradually as the incubation time increased (Figures 3A,B). All of the hydrogels degraded by 20–33% after 7 days, 50–63% after 14 days, 54–68% after 21 days, and 66–78% after 28 days (Figure 3A). When the hydrogels were submerged in PBS in the presence of hyaluronidase, the weight loss by degradation was significantly higher than that in PBS solution (Figure 3B). All of the hydrogels degraded by 82–93% after 28 days; this fast degradation rate was related to the β-elimination caused by hyaluronidase (Zhu et al., 2018b). Compared with OHA/HA-ADH, the OHA/HA-ADH/O-HACC and OHA/HA-ADH/N-HACC hydrogels shown a lower weight loss ratio. The results indicated that the addition of O-HACC and N-HACC effectively improves the enzymatic stability of the hydrogels by increasing the formation of dynamic chemical bonds (Schiff base linkage between -NH_2_ and -CHO) (Qu et al., 2018). Therefore, the developed hydrogels could prolong the usage period and reduce replacement frequency. The MIC values of the O-HACC and N-HACC obtained were tested and are shown in Table S1. The results showed that the MIC values of O-HACC and N-HACC against gram-negative bacteria *E. coli* were 6.25 and 0.039 mg/mL and against gram-positive bacteria *S. aureus* were 24.2 and 0.078 mg/mL, respectively. In addition, the antibacterial effect of the O-HACC and N-HACC series against *E. coli* was stronger than that against *S. aureus*.

### *In vitro* Biocompatibility of the Hydrogels

**Figure 4:**
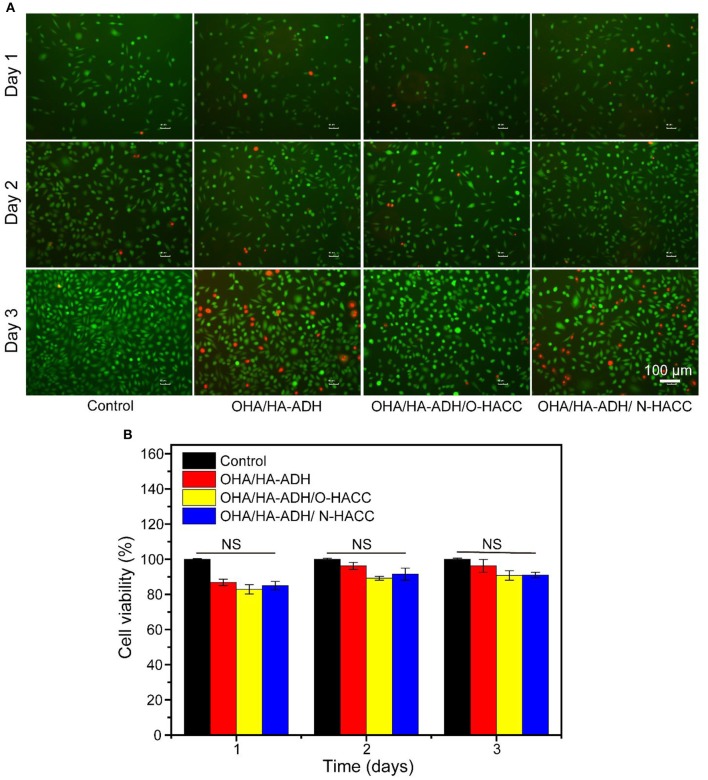
*In vitro* biocompatibility evaluation of the hydrogels. **(A)** Fluorescence images of L929 cells after live/dead staining at day 1, day 2, and day 3. Green fluorescence indicates living cells, and red fluorescence indicates dead cells. **(B)** Cell viability of L929 cells at different culture times according to CCK-8 assay.

On the basis that the developed hydrogels had good physical properties and antibacterial activity, it was necessary to determine their biocompatibility, which is a prerequisite for well-designed biomedical materials (Wu et al., 2016; Cai et al., 2018). The cell viability ratio was evaluated by culturing murine-derived cell line L929 fibroblast cells with the leach liquor of the hydrogels. TCP and OHA/HA-ADH hydrogel without O-HACC and N-HACC were used as the control groups. It was clear that continuous increases in cell intensity from days 1 to 3 were observed for all groups, which indicated continuous proliferation of L929 fibroblast cells (Figure 4A). There were a large number of alive cells (green) and few dead cells (red) in all hydrogel groups, and they showed no obvious difference from the control groups, which indicated the innate biocompatibility of the hydrogels. It is worth noting that the cell densities of the OHA/HA-ADH/O-HACC and HA/HA-ADH/N-HACC groups at predetermined time points were slightly lower than those of the OHA/HA-ADH hydrogel A and TCP groups.

The cell viability rate of L929 fibroblast cells on the hydrogel groups was further quantitatively examined in accordance with the CCK-8 assay, as shown in Figure 4B. For the first and second days, the cell viability of all of the hydrogel groups was comparable to the control groups. On the third day, the OHA/HA-ADH/N-HACC and OHA/HA-ADH/O-HACC groups exhibited slightly lower cell viability than the OHA/HA-ADH hydrogel and TCP groups because of the interaction between N-HACC and O-HACC, which have a positive charge, and the cells, but it still comparable with the control group (Kowapradit et al., 2011). This excellent biocompatibility can be related to the dynamic chemical bond (Schiff base linkage) of the hydrogels. All of the results confirmed that the addition of N-HACC or O-HACC to an OHA/HA-ADH hydrogel resulted in satisfactory biocompatibility that was favorable for L929 cell growth and proliferation.

### Bacterial Growth on the Hydrogel Surface and SEM Imaging

**Figure 5:**
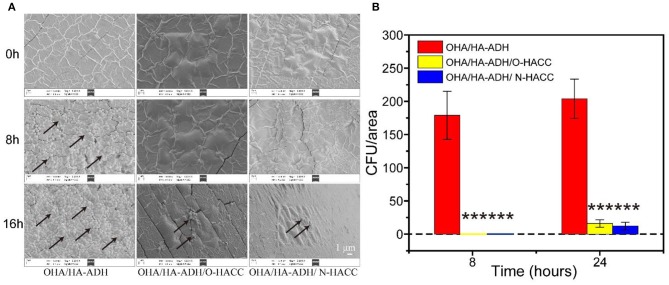
**(A)** SEM images demonstrating the antibacterial activity of OHA/HA-ADH, OHA/HA-ADH/O-HACC, and OHA/HA-ADH/N-HACC hydrogels. **(B)** Quantitative analysis of the number of bacteria in each image area. ***p < 0.001.

Besides serving as a barrier to protect wound tissues from external bacterial infections, hydrogels with inherent antimicrobial properties will be particularly attractive because they can prevent wound infections and promote the healing process (Gopinath et al., 2004). *S. aureus* was selected as a representative bacterium to evaluate the antimicrobial effect of the developed hydrogels. The inhibition of the growth of *S. aureus* by the developed hydrogels was assessed by measuring the number of *S. aureus* on the surface of the hydrogels. Bacterial growth on the hydrogels was observed using an SEM. It was clear that the OHA/HA-ADH hydrogel, which lacked antimicrobial material, had a large number of *S. aureus* attached to it, whereas *S. aureus* had significantly inhibited growth and was killed by OHA/HA-ADH/O-HACC and HA/HA-ADH/N-HACC hydrogels (Figure 5A). In addition, at 8 h, the number of *S. aureus* on OHA/HA-ADH was calculated to be about 179 CFU/area. However, the number of bacteria on OHA/HA-ADH/O-HACC and HA/HA-ADH/N-HACC hydrogels was 0 CFU/area for *S. aureus* (Figure 5B). At 24 h, the bacterial growth in the OHA/HA-ADH hydrogel increased significantly, with the bacteria number calculated to be 204 CFU/area, whereas the number of bacteria in OHA/HA-ADH/O-HACC and HA/HA-ADH/N-HACC hydrogels was only 16 and 12 CFU/area, respectively. The results indicated that *S. aureus* on the surface of the hydrogels were significantly inhibited or killed by the antimicrobial hydrogels (Peng et al., 2010).

### The Hydrogels Promote Seawater-Immersion Wound Healing

**Figure 6:**
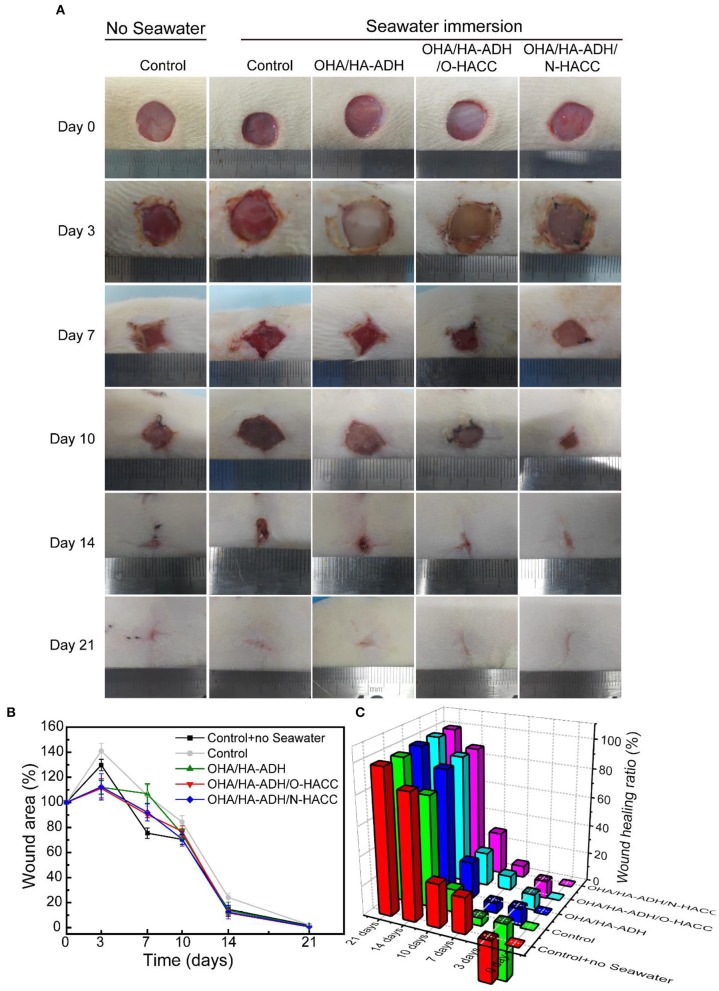
**(A)** Photographs of wounds caused in the control group with or without seawater immersion and OHA/HA-ADH, OHA/HA-ADH/O-HACC, and OHA/HA-ADH/N-HACC groups with seawater immersion. **(B)** Percentage change in wound area, presented as mean ± SD. **(C)** Wound healing ratio over a 21-day period.

The effect of the hydrogels on wound healing was further evaluated *in vivo* by using an animal wound model with seawater immersion. Figure 6A shows representative images of the wound surface on days 0, 3, 7, 10, 14, and 21 for each group. The wound area in all five groups became larger before 3 days, and obvious inflammation was observed in the control and OHA/HA-ADH groups with seawater immersion at day 3. Though the wound bed was infected, the wound area in the OHA/HA-ADH, OHA/HA-ADH/O-HACC, and OHA/HA-ADH/N-HACC groups became smaller with increasing time after 3 days, scab formation was observed in all groups at day 7. At day 14, the wounds of the control and OHA/HA-ADH groups had not healed well, whereas the wounds of the OHA/HA-ADH/O-HACC and OHA/HA-ADH/N-HACC groups had healed significantly. It was found that the OHA/HA-ADH/O-HACC and OHA/HA-ADH/N-HACC hydrogels significantly promoted wound healing, which could be ascribed to the combined effects of the inherent antibacterial performance of O-HACC and N-HACC and the moist wound bed provided by the OHA/HA-ADH hydrogel dressing.

The wound area percentage of the different groups at days 0, 3, 7, 10, 14, and 21 is shown in Figure 6B. The control group without seawater immersion showed the lowest wound area percentage of the groups. At days 3, 7, 10, and 14, the wound area percentages of the OHA/HA-ADH/O-HACC and OHA/HA-ADH/N-HACC groups were significantly lower than those of the control group and OHA/HA-ADH group with seawater immersion and were comparable with seawater immersion, which indicated that the addition of the antibacterial material (O-HACC and N-HACC) could induce bacterial death and prevent further infection. The same trend is presented through a more intuitive three-dimensional view of the wound healing rate in Figure 6C. In the results, OHA/HA-ADH/O-HACC and OHA/HA-ADH/N-HACC hydrogels can be seen to accelerate and promote wound closure and re-epithelialization on the seawater-immersed wound infection of full-thickness skin wounds. Moreover, the wound-healing effect of the OHA/HA-ADH/N-HACC group was better than that of OHA/HA-ADH/O-HACC, which might be attributable to the antibacterial effect of N-HACC being stronger than that of O-HACC.

### Number of Bacteria on Wound

**Figure 7:**
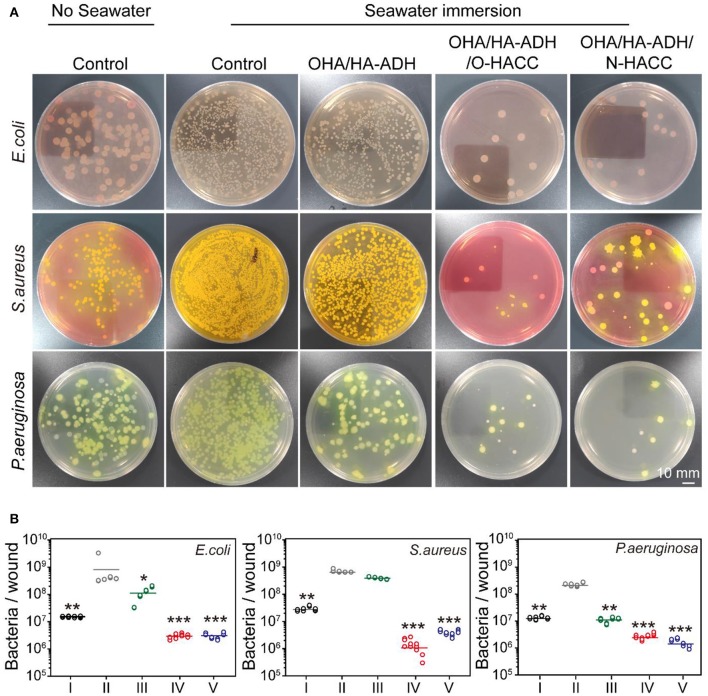
**(A)** *E. coli, S. aureus*, and *P. aeruginosa* colonies from tissues formed on agar plates. A red ring-like morphology precipitating on VRBA-MUG selection medium means *E. coli*, a yellow-gold colony morphology on Mannitol salt agar indicates *S. aureus*, and a green-yellow colony morphology on *P. aeruginosa* selection medium represents *P. aeruginosa*. **(B)** Quantitative analysis of the number of bacteria remaining in the wound area. *p < 0.05, **p < 0.01, ***p < 0.001.

The bacterial count on seawater-immersed wound infections of full-thickness wounds was determined at day 3. The bacteria from the wound site tissues were cultured on three selected agar media for 24 h and were then counted for further analysis (Figure 7). The results demonstrated that the number of bacteria on the seawater immersed wounds in the control group were approximately 6.2 × 10^8^ CFU/wound for *E. coli*, 6.8 × 10^8^ CFU/wound for *S. aureus*, and 2.3 × 10^8^ CFU/wound for *P. aeruginosa*. However, the number of bacteria was significantly reduced in the OHA/HA-ADH/O-HACC group (~10^6^ CFU/wound for *E. coli*, 1.3 × 10^6^ CFU/wound for *S. aureus*, and 2.7 × 10^6^ CFU/wound for *P. aeruginosa*) and the OHA/HA-ADH/N-HACC group (3.0 × 10^6^ CFU/wound for *E. coli*, 3.9 × 10^6^ CFU/wound for *S. aureus*, and 1.6 × 10^6^ CFU/wound for *P. aeruginosa*) at day 3. The results demonstrated that the bacteria in the wound site were inhibited or killed by the antimicrobial hydrogels. In addition, the antimicrobial hydrogels could accelerate skin wound closure and healing by reducing the bacterial counts in seawater-immersed wounds of rats.

### Histological Observations

**Figure 8:**
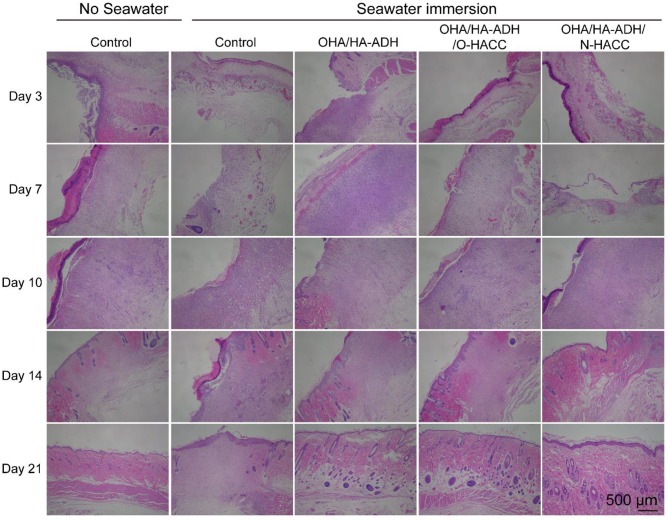
Representative images with H&E staining of the effect of seawater immersion on wound sections at day 3, day 7, day 10, day 14, and day 21.

The histologic structures of the regenerated dermis were stained with H&E to evaluate wound healing progress. The degrees of epithelial closure and granulation tissue formation in the different treatment groups were very pronounced (Figure 8). At day 3, a clear newly formed squamous epithelial layer was observed in OHA/HA-ADH/O-HACC and OHA/HA-ADH/N-HACC hydrogels, whereas the OHA/HA-ADH hydrogels and the control groups with seawater immersion showed an incomplete epidermal structure and a thinner dermal layer. In addition, obvious inflammatory cell infiltration was present in the wounds of the control group with seawater immersion on day 7, whereas the number of inflammatory cells on the OHA/HA-ADH/O-HACC and OHA/HA-ADH/N-HACC hydrogel groups was negligible (locally enlarged images of the tissue sections are shown in Figure S2). Remarkably, thick and well-formed granulation tissue was apparent in the OHA/HA-ADH/O-HACC and OHA/HA-ADH/N-HACC hydrogel-treated group on day 10, indicating that the antimicrobial hydrogels could promote rapid wound healing. At day 14, the epidermis of the new granulation tissue was integrated and thick in both the OHA/HA-ADH/O-HACC and OHA/HA-ADH/N-HACC hydrogel groups. At day 21, the OHA/HA-ADH/O-HACC and OHA/HA-ADH/N-HACC hydrogel groups developed some newly formed hair follicles in the center of the wound, which were not seen in the control group with seawater immersion. However, with respect to the microstructure of regenerated tissues, a more complete re-epithelialization and tight junction between epidermis and dermis was found in the antimicrobial hydrogel groups, which were significant for functional and scar-free tissue recovery.

### Collagen Deposition

**Figure 9:**
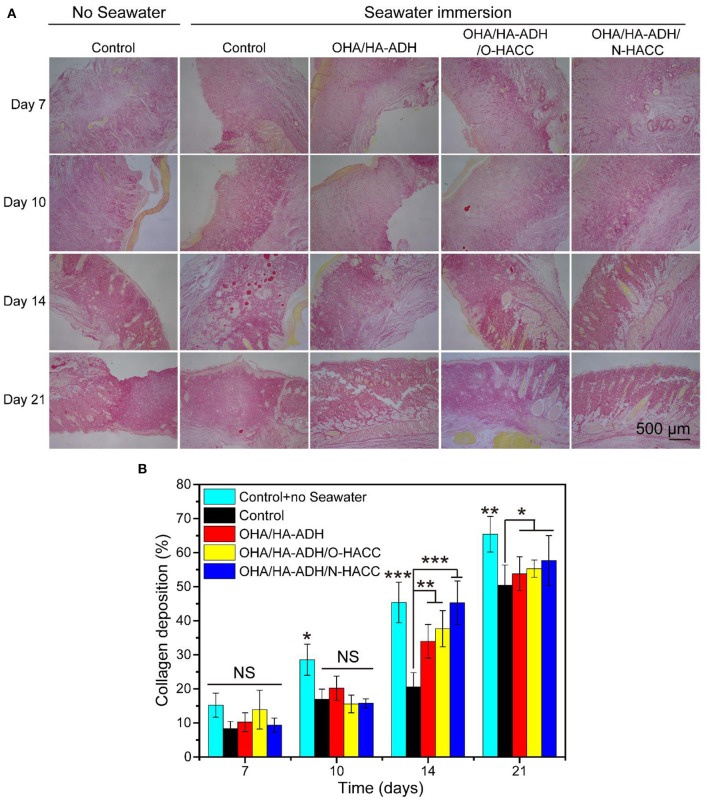
Collagen deposition in wound sites. **(A)** Representative images of Sirius red staining in the control group with or without seawater immersion and the OHA/HA-ADH, OHA/HA-ADH/O-HACC, and OHA/HA-ADH/N-HACC groups with seawater immersion. **(B)** Quantitative analysis of collagen deposition in wound tissue. *p < 0.05, **p < 0.01, ***p < 0.001.

In order to gain more insight into the granulation tissue, collagen deposition and maturation were further assessed in each group by Picrosirius red staining (Figure 9A). Ipp 6.0 software was used to quantify the collagen deposition in the wound sites by calculating the intensity of the red areas in tissue area (Figure 9B). After 14 days of treatment, it can be seen that the OHA/HA-ADH/O-HACC and OHA/HA-ADH/N-HACC hydrogel groups not only had nearly twice as much collagen deposition as the other groups, as reflected by the Picrosirius red staining, but also had the most organized fibrous structure (local enlarged images of the wound sections are shown in Figure S3). In addition, at day 21, more mature fibers with regular orientation and distribution were seen in the wound sites treated with OHA/HA-ADH/O-HACC and OHA/HA-ADH/N-HACC as compared to those treated with the control with seawater immersion. With further quantitative analysis, the collagen contents in the wounds treated with OHA/HA-ADH/O-HACC and OHA/HA-ADH/N-HACC hydrogel were significantly higher than those of the OHA/HA-ADH hydrogel and control group without seawater (*p* < 0.01) and the control group with seawater (*p* < 0.001) at day 14. The enhanced collagen deposition could be attributed to the HA (which promotes collagen synthesis in fibroblasts) and the antibacterial properties of O-HACC or N-HACC (Kisiel et al., 2013; Hu et al., 2019).

### Immunohistochemistry

**Figure 10:**
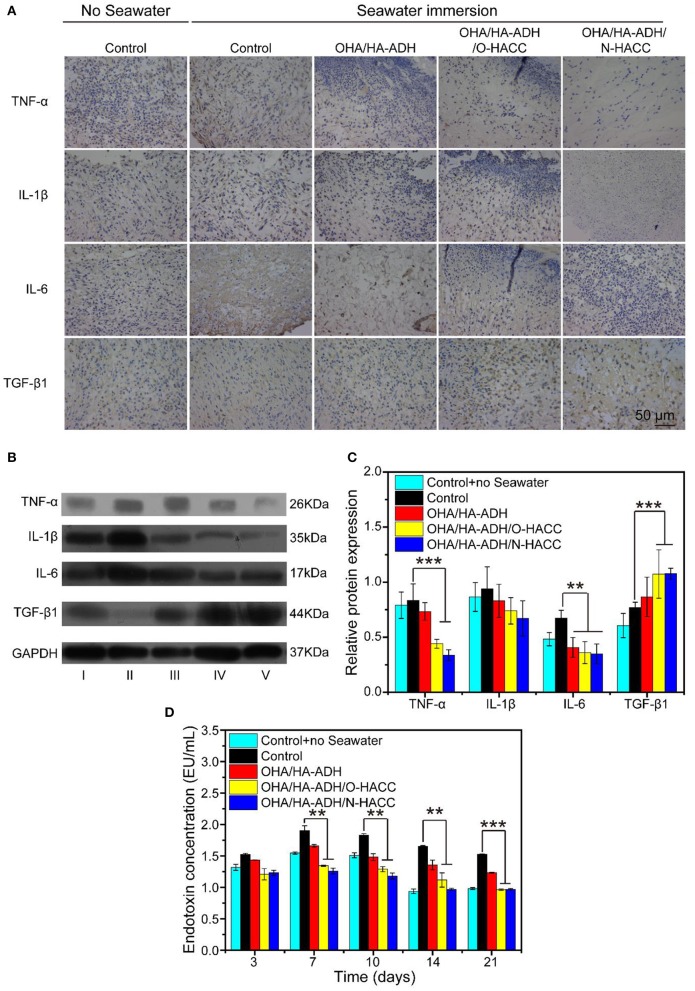
**(A)** Representative images of immunohistochemistry pro-inflammatory factors (TNF-α, IL-1β, and IL-6) and anti-inflammatory factors (TGF-β1) of wound sections. **(B)** Western blot analyses were performed for TNF-α, IL-1β, IL-6, and TGF-β1. **(C)** Quantitative analysis of inflammatory factor. **(D)** Comparison of serum endotoxin concentration (EU/mL) in the different groups. **p < 0.01, ***p < 0.001.

The mechanism of the prepared hydrogel-promoted wound healing in the rat wound with seawater immersion model was explored by performing immunohistochemistry with pro-inflammatory factors (TNF-α, IL-1β, and IL-6) and anti-inflammatory factors (TGF-β1) in the wound (Figure 10A). The OHA/HA-ADH/O-HACC and OHA/HA-ADH/N-HACC hydrogel treatment decreased the expression level of pro-inflammatory factors (TNF-α, IL-1β, and IL-6) at day 7. On the other hand, the expression level of the anti-inflammatory factors (TGF-β1) was enhanced much more in the OHA/HA-ADH/O-HACC and OHA/HA-ADH/N-HACC hydrogel groups than in the OHA/HA-ADH hydrogel and control groups. The results indicated that OHA/HA-ADH/O-HACC and OHA/HA-ADH/N-HACC hydrogels reduced the expression of pro-inflammatory factors and increased that of anti-inflammatory factors in a wound with seawater immersion, which was important for the wound healing process. The Western blot analysis of TGF-β1, TNF-α, IL-1β, and IL-6 further supports these results, as less pro-inflammatory factors and more anti-inflammatory factors occurred in the antimicrobial hydrogel-treated wounds compared to the other groups (Figures 10B,C), which could be attributed to O-HACC and N-HACC being efficient inherently antibacterial materials capable of reducing infection (Qu et al., 2019).

### Endotoxin Concentration

More specifically, the serum endotoxin levels of five groups were substantially elevated in the OHA/HA-ADH/O-HACC and OHA/HA-ADH/N-HACC groups compared to the control group with seawater (Figure 10D). The endotoxin concentration of the control group without seawater was about 1.0–1.5 EU/mL. However, the highest endotoxin concentration of the control group with seawater was about 1.5 EU/mL at day 3, 1.9 EU/mL at day 7, 1.8 EU/mL at day 10, 1.6 EU/mL at day 14, and 1.5 EU/mL at day 21. In addition, there was a significant decrease in endotoxin concentration in the OHA/HA-ADH/O-HACC and OHA/HA-ADH/N-HACC group compared to the control group with seawater (*P* < 0.05 at days 7, 10, and 14 and *P* < 0.001 at day 21), which was related to the bacteria numbers of their corresponding groups.

## Conclusion

The aim of the present work was to develop biocompatible hydrogel wound dressings with inherent antibacterial properties. OHA/HA-ADH hydrogels with the addition of quaternized chitosan, OHA/HA-ADH/O-HACC and OHA/HA-ADH/N-HACC, were successfully prepared by dynamic Schiff base linkage. These hydrogels exhibited stable rheological properties, appropriate swelling properties, water vapor transmission rate, and biodegradability, inherent antibacterial properties, and good biocompatibility, meaning that they could effectively promote the wound healing process. Furthermore, the hydrogels accelerated the healing process in a seawater-immersed full-thickness skin defect model *in vivo*, as well as exhibiting excellent reepithelialization and greater granulation tissue thickness, higher-density collagen deposition, a smaller number of bacteria, a lower endotoxin level, and more balanced inflammatory infiltration than control. In particular, by reducing wound healing process-related pro-inflammatory factors (TNF-α, IL-1β, and IL-6) and upregulating anti-inflammatory factors (TGF-β1) in wound, the OHA/HA-ADH/O-HACC and OHA/HA-ADH/N-HACC antibacterial hydrogels presented the best wound healing effect among all groups. All results demonstrated that OHA/HA-ADH/O-HACC and OHA/HA-ADH/N-HACC represent promising antimicrobial hydrogel wound dressings for wound healing application, especially for open and infected traumas, due to their good bioactivity and inherent antibacterial properties.

## Data Availability Statement

All datasets generated for this study are included in the article/Supplementary Material.

## Ethics Statement

This animal study was reviewed and approved by the Institutional Animal Care and Use Committee (IACUC) of the General Hospital of the Southern Theater Command of the PLA.

## Author Contributions

RG and BC designed the experiments. XW and PX conducted most of the experiments and analyzed the data. ZY helped with cell experiments. QF and LF helped with animal experiments and *in vivo* characterization. XW, PX, RG, and BC wrote the manuscript.

### Conflict of Interest

LF was employed by company Beogene Biotech Co. The remaining authors declare that the research was conducted in the absence of any commercial or financial relationships that could be construed as a potential conflict of interest.
