# Supplementary material for: Preparation of Antimicrobial Hyaluronic Acid/Quaternized Chitosan Hydrogels for the Promotion of Seawater-Immersion Wound Healing
Source: Front Bioeng Biotechnol. 2019 Dec 10;7:360. doi: 10.3389/fbioe.2019.00360 (PMC6914676; doi:10.3389/fbioe.2019.00360)
Supplement: Supplementary file 1 [file Data_Sheet_1.docx]

**Supporting Information**

**Preparation of antimicrobial hyaluronic acid/quaternized chitosan hydrogels for the promotion of seawater immersion wound healing**

Xinlu Wang ^a, b 1^, Pengcheng Xu ^b 1^, Zexin Yao ^b, c^, Qi Fang ^a^, Longbao Feng ^d^, Rui Guo ^e *^, Biao Cheng ^b *^

^a^ The First Clinical Hospital of Guangzhou Medical University, Guangzhou 510120, China

^b^ Department of Plastic Surgery, General Hospital of Southern Theater Command, PLA, Guangzhou 510010, China

^c^ Guangdong Pharmaceutical University, Guangzhou 510120, China

^d^ Beogene Biotech (Guangzhou) CO., LTD, Guangzhou 510663, China

^e^ Key Laboratory of Biomaterials of Guangdong Higher Education Institutes, Guangdong Provincial Engineering and Technological Research Center for Drug Carrier Development, Department of Biomedical Engineering, Jinan University, Guangzhou 510632, China

^1^ These authors contributed equally to this work.

* Corresponding author: chengbiaocheng@163.com; guorui@jnu.edu.cn

Tel/Fax: +86-20-85222942

**Supplementary Table 1 Antimicrobial MIC (mg/mL) of quaternary ammonium chitosan.**

| Polymer | **(MIC, mg/mL)** | | |
| --- | --- | --- | --- |
|  | *E. coli* | *S. aureus* | |
| O-HACC | 6.25 | | 24.2 |
| N-HACC | 0.039 | | 0.078 |

**
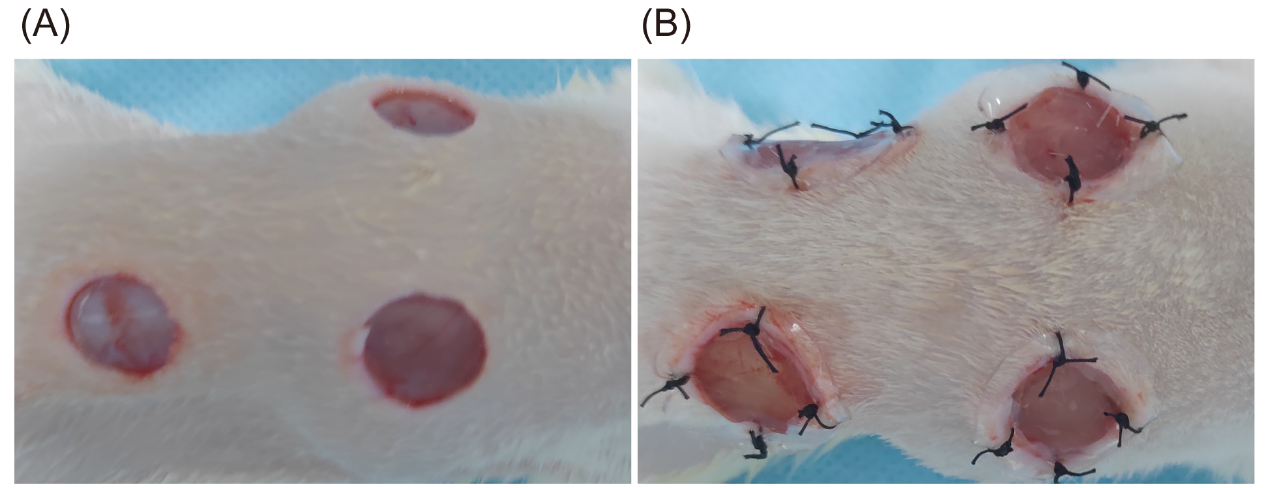
**

**Figure S1** (A) Full-thickness wound models on skin of SD rat with seawater immersion. (B) Wounds were sewn up with the hydrogels using silicon film.


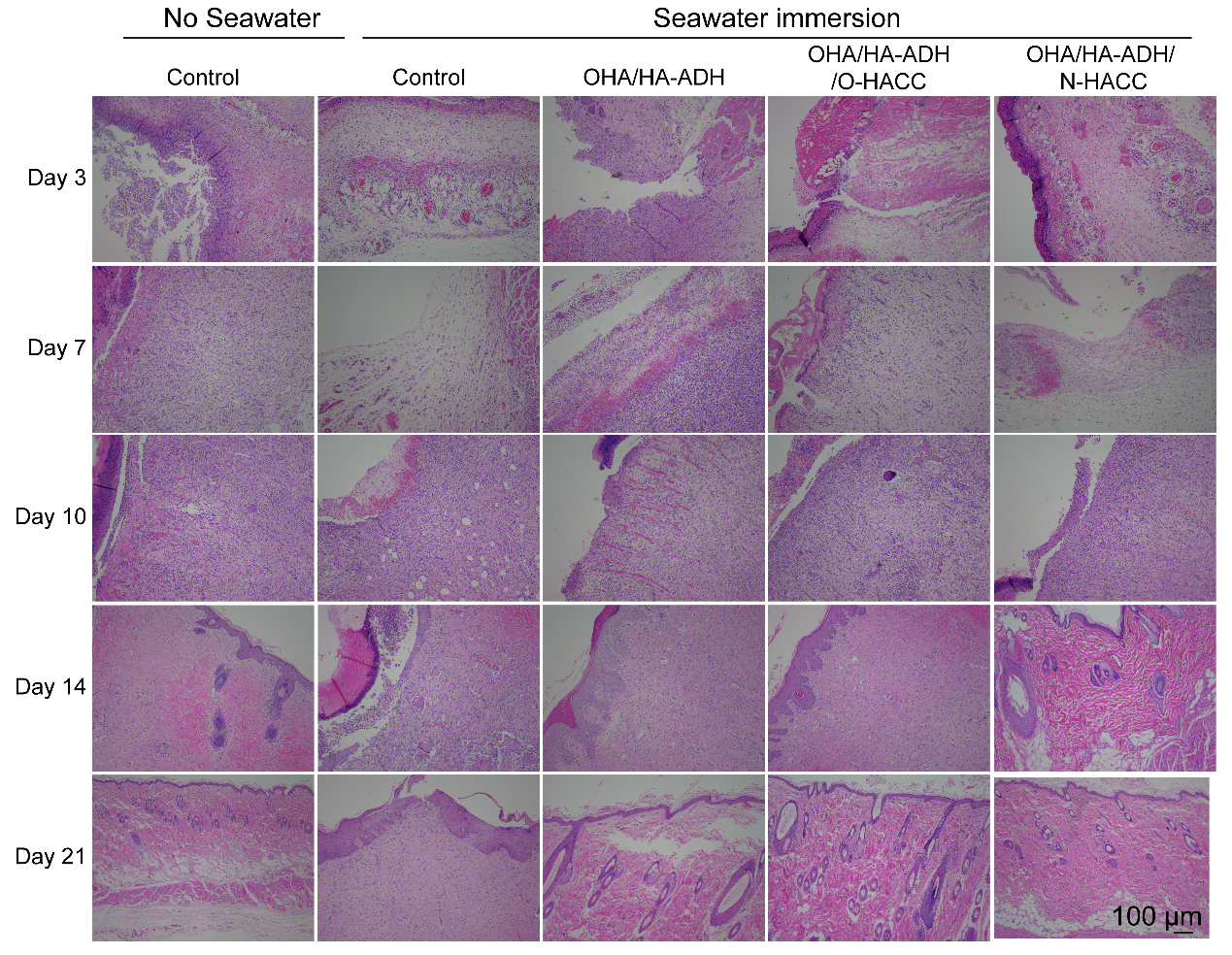


**Figure S2** Locally enlarged images of H&E at day 3, day 7, day 10, day 14, and day 21.


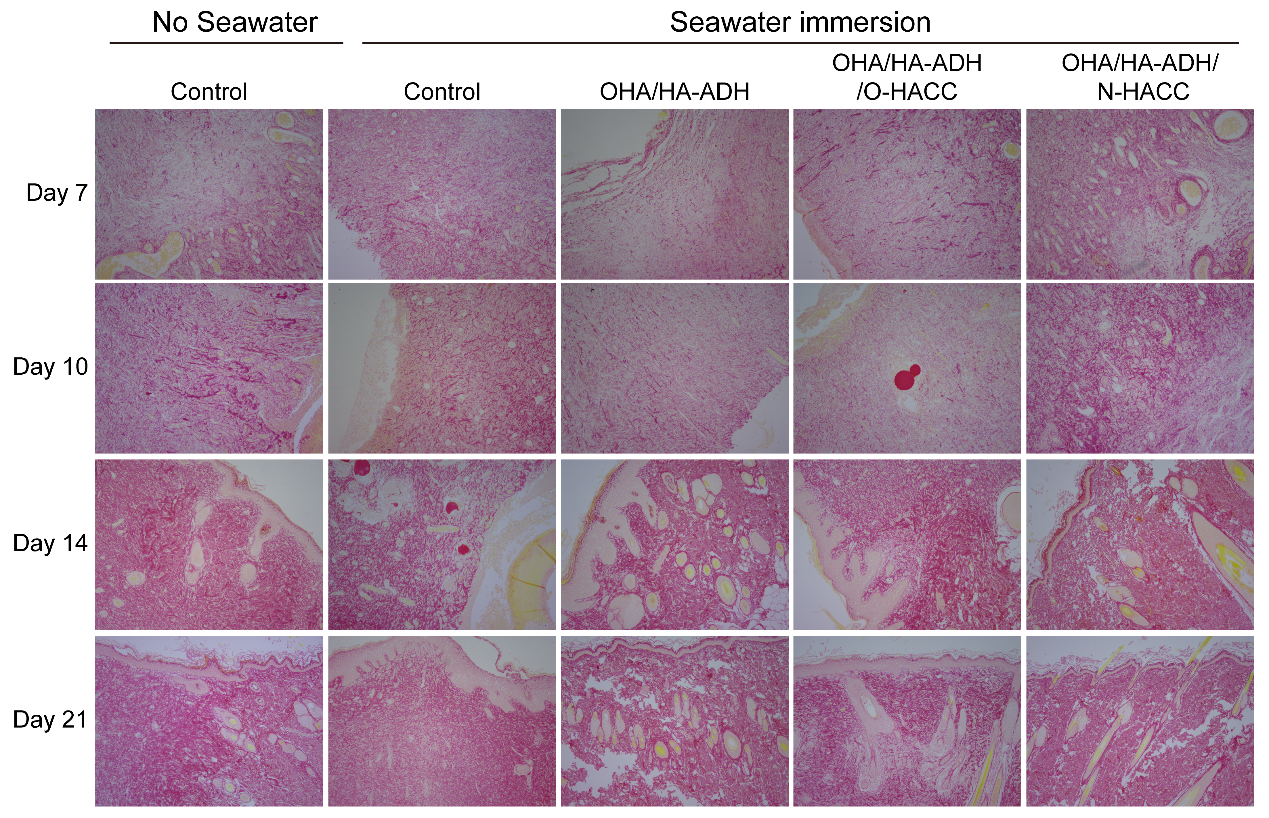


**Figure S3** Locally enlarged images of Sirius red staining at day 7, day 10, day 14, and day 21.
